# Supplementary material for: Delay in Seeking Medical Attention and Diagnosis in Chinese Melanoma Patients: A Cross-Sectional Study
Source: Int J Environ Res Public Health. 2022 Nov 12;19(22):14916. doi: 10.3390/ijerph192214916 (PMC9690906; doi:10.3390/ijerph192214916)
Supplement: Supplementary file 1 [file ijerph-19-14916-s001.zip › ijerph-2035364-supplementary.pdf]

Supplementary Material

Supplementary Material S1

A survey on the health behaviour of patients with cutaneous melanoma

Hello, dear patients! In order to better understand the information of your disease and your medical process, and to provide you with better services in the future, we are carrying out this questionnaire, hoping to get your real thoughts and valuable opinions. Thank you for your support and cooperation!

Q1: what is your date of birth?

Q2: what is your gender?
-male
-female

Q3: What is your education level?
- Primary school and lower
- Junior middle school
- Senior middle school
- College / university
- Postgraduate or above

Q4: What is your annual household income?
- Under 10,000 CNY
- 10,000-29,000 CNY
- 30,000-49,000 CNY
- 50,000-99,000 CNY
- Above 100,000 CNY

Q5: What is your marital status?
- Unmarried
- married
- divorced
- Widowed

Q6: Which of the following are your onset sites? (Indeterminate multiple-choice questions)
- Soles of the feet
- Fingernail
- Toenail
- Palm
- Extremities

- Trunk
- Face
- Scalp
- Neck
- Vulva
- Others, please specify

Q7: Have you ever been diagnosed by a doctor with another malignancy?
- Negative
- Positive, please specify

Q8: Have you ever visited a doctor for a mole, dark spot, black lump or other suspicious skin lesion?
- Negative
- Positive

Q9: What was the interval between the time you noticed the suspicious lesion and the time you first sought medical help? (fill in the blank)
Year: Month:

Q10: Where did you first visit for this disease?
- Level III General Hospital
- Level II Specialist Hospitals
- Specialist hospitals (e.g. oncology hospitals)
- Primary Hospitals
- Other, please specify

Q11: What was the time interval between first seeking medical help and the diagnosis of melanoma? (fill in the blank)
Year: Month:

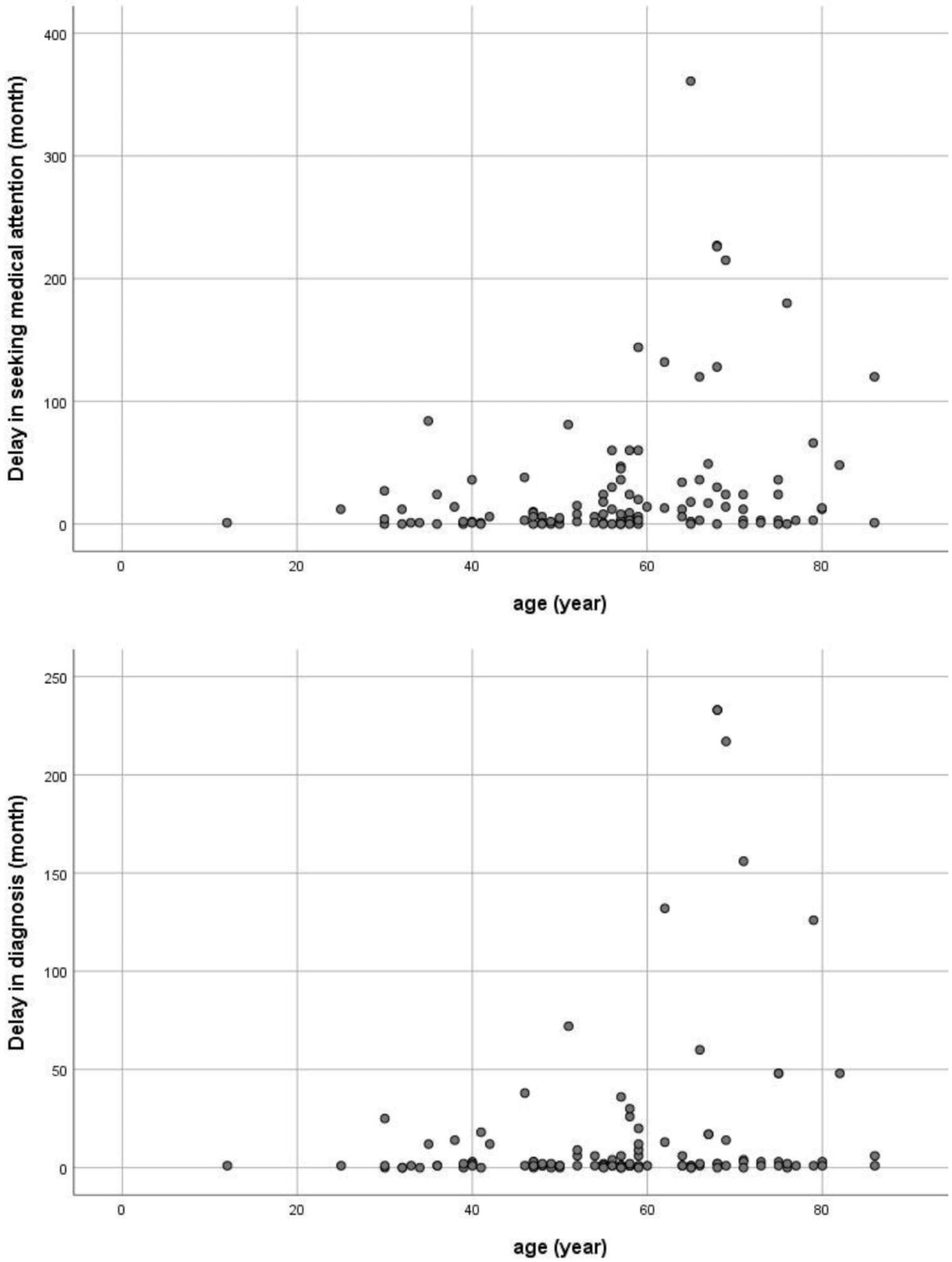

Figure S1. Scattergrams of DSMA, DD, and age.

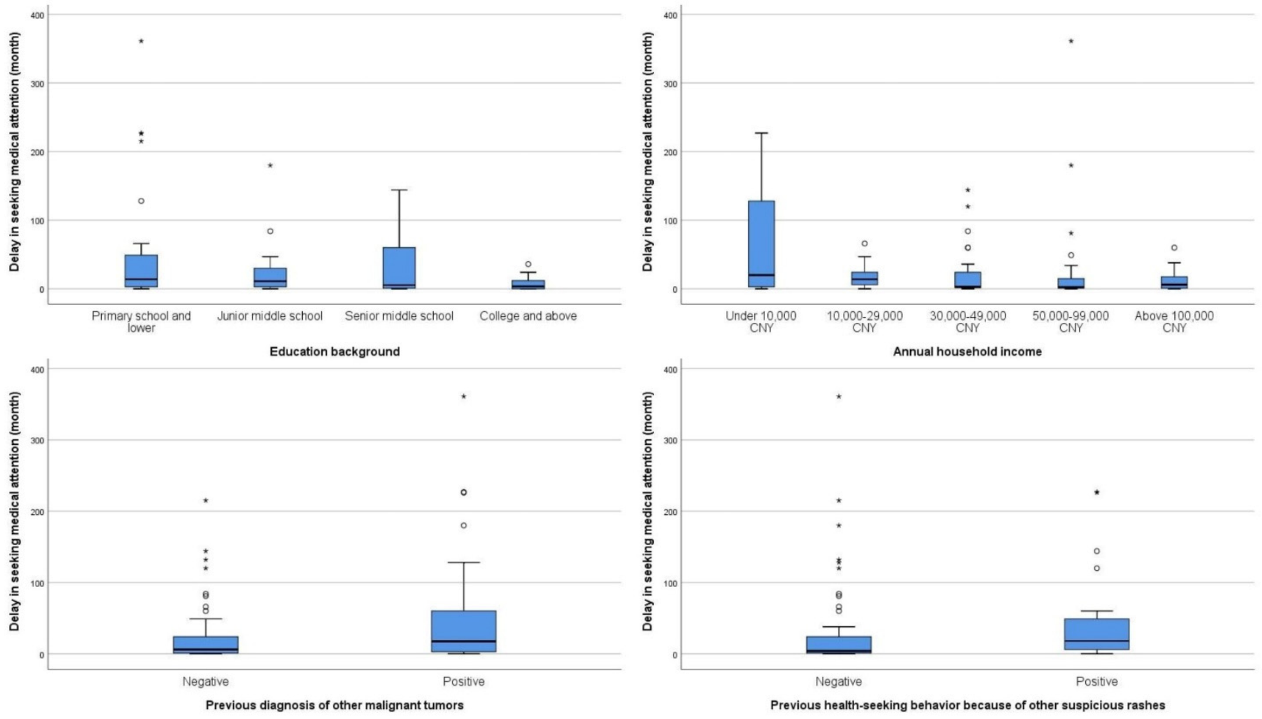

Figure S2: Box-plots of DSMA with education background, annual household income, previous diagnosis of other malignant tumors, and previous health-seeking behavior because of other suspicious lesions.

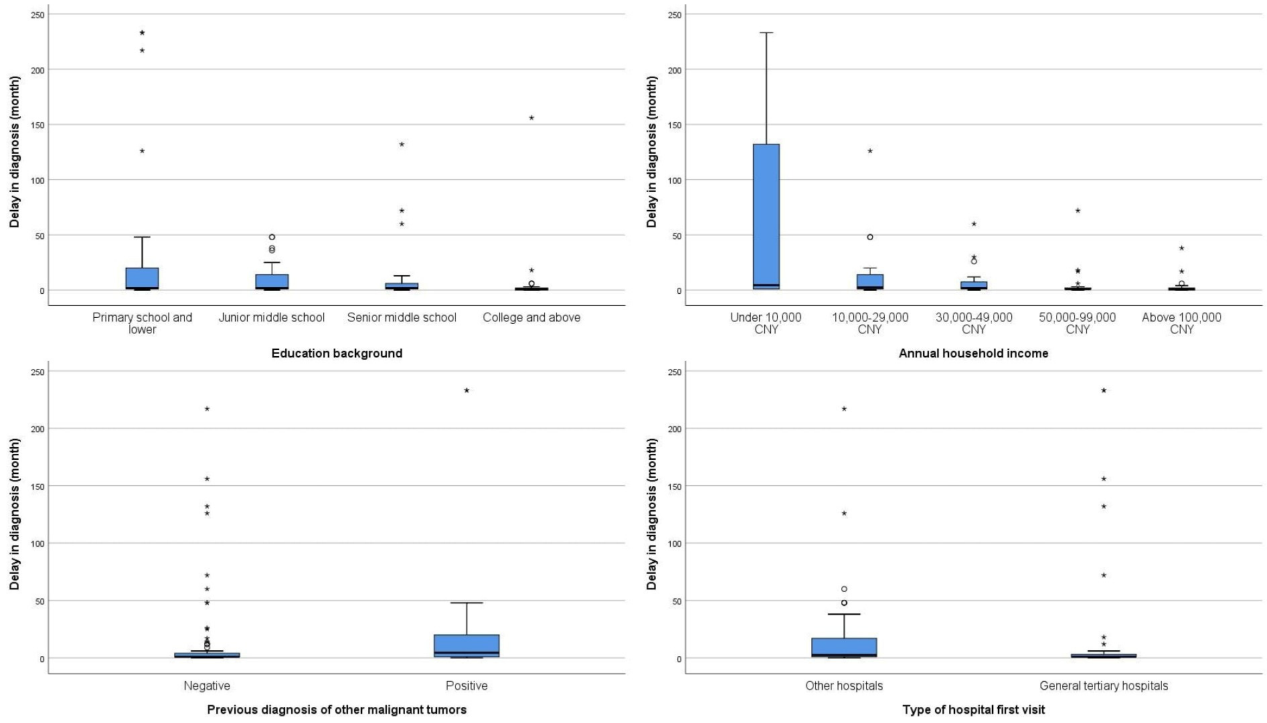

Figure S3: Box plots of DD with education background, annual household income, previous diagnosis of other malignant tumors, and type of hospital first visit.

Table S1: Gamma regression with log-link of DSMA and DD.

Variables	DSMA		DD	
	OR (95%CI)	*P*	OR (95%CI)	*P*
Age	1.02 (1.00- 1.05)	**0.08**	1.02 (1.00- 1.05)	**0.06**

Sex	0.78 (0.43-1.41)	0.41	0.80 (0.42-1.51)	0.50
Educational background				
Junior high school and lower	ref		ref	
Senior high school and above	0.69 (0.35-1.36)	0.29	1.43 (0.62-3.32)	0.40
Annual household income				
Under 29000 CNY	ref		ref	
30000-99000 CNY	0.83 (0.41-1.70)	0.61	0.23 (0.11-0.49)	**<0.01**
Above 100000 CNY	0.62 (0.28-1.41)	0.25	0.10 (0.05-0.24)	**<0.01**
Previous diagnosis of other malignant tumors				
Negative	ref		ref	
Positive	1.99 (0.96-4.12)	**0.06**	2.49 (1.17-5.27)	**0.02**
Previous health-seeking behavior because of other suspicious lesions				
Negative	ref			
Positive	1.81 (0.93-3.54)	**0.08**		
Type of hospital first visit				
Other hospitals			ref	
General tertiary hospitals			0.70 (0.32-1.52)	0.36

Bold text denotes significance ($P < 0.10$).
DSMA, delay in seeking medical attention; DD, delay in diagnosis.
OR: odds ratio
